# Synthetic Data in Healthcare and Drug Development: Definitions, Regulatory Frameworks, Issues

**DOI:** 10.1002/psp4.70021

**Published:** 2025-04-07

**Authors:** Giuseppe Pasculli, Marco Virgolin, Puja Myles, Anna Vidovszky, Charles Fisher, Elisabetta Biasin, Miranda Mourby, Francesco Pappalardo, Saverio D'Amico, Mario Torchia, Alexander Chebykin, Vincenzo Carbone, Luca Emili, Daniel Roeshammar

**Affiliations:** ^1^ InSilicoTrials Technologies S.p.A. Trieste Italy; ^2^ InSilicoTrials Technologies B.V. s‐Hertogenbosch the Netherlands; ^3^ Medicines and Healthcare products Regulatory Agency London UK; ^4^ Unlearn.AI San Francisco California USA; ^5^ Centre for IT & IP Law (CiTiP), KU Leuven Leuven Belgium; ^6^ Centre for Health, Law, and Emerging Technologies (HeLEX), Faculty of Law University of Oxford Oxford UK; ^7^ Department of Drug and Health Sciences University of Catania Catania Italy; ^8^ Humanitas Clinical and Research Center‐IRCCS Milan Italy; ^9^ Train s.r.l. Milan Italy

**Keywords:** drug development, external control arms, generative AI, medical devices, real‐world data, regulatory framework, synthetic data

## Abstract

With the recent and evolving regulatory frameworks regarding the usage of Artificial Intelligence (AI) in both drug and medical device development, the differentiation between data derived from observed (‘true’ or ‘real’) sources and artificial data obtained using process‐driven and/or (data‐driven) algorithmic processes is emerging as a critical consideration in clinical research and regulatory discourse. We conducted a critical literature review that revealed evidence of the current ambivalent usage of the term “synthetic” (along with derivative terms) to refer to “true/observed” data in the context of clinical trials and AI‐generated data (or “artificial” data). This paper, stemming from a critical evaluation of different perspectives captured from the scientific literature and recent regulatory endeavors, seeks to elucidate this distinction, exploring their respective utilities, regulatory stances, and upcoming needs, as well as the potential for both data types in advancing medical science and therapeutic development.

**Abbreviations**
AI: Artificial Intelligence
CBER: Center for Biologics Evaluation and Research within FDA
CDER: Center for Drug Evaluation and Research within FDA
CDRH: Center for Devices and Radiological Health within FDA
CM&S: Computer Modeling and Simulation
CPRD: Clinical Practice Research Datalink
DGA: Data Governance Act
DMs: Diffusion Models
ECA: External Control Arm
EHDS: European Health Data Space
EHR: Electronic Health Record
EMA: European Medicines Agency
ER: Exposure‐Response model
FDA: US Food and Drug Administration
GANs: Generative Adversarial Networks
GDPR: General Data Protection Regulation
ICH: International Conference on Harmonization
ISPE: International Society for Pharmacoepidemiology
IVDR: In Vitro Diagnostic Medical Devices Regulation
MHRA: Medicines and Healthcare products Regulatory Agency
ML: Machine Learning
ODE: Ordinary Differential Equation
PBPK: Physiologically based Pharmacokinetic model
PD: Pharmacodynamic
PK: Pharmacokinetic
popPK: Population Pharmacokinetic model
popPKPD: Population Pharmacokinetic/Pharmacodynamic model
QSP: Quantitative Systems Pharmacology
RCTs: Randomized Controlled Trials
RWD: Real‐World Data
SCAs: Synthetic Control Arms
US: United States
VAEs: Variational Autoencoders
VCAs: Virtual Control Arms

## 1 Introduction

In the face of rapidly growing data challenges in the global healthcare sector, such as privacy concerns, confidentiality, data fragmentation, validity questions, interoperability, and generalizability issues, synthetic data are stepping forward as a potential source of innovation. The European Health Data Space (EHDS) proposal (entered into force in March 2025 and published in the European Official Journal) by the European Commission aims to establish a unified data market, leveraging health data for care delivery, research, and policy development [1]. Synthetic data may be a key element in this initiative, as they can facilitate scientific advancement without compromising data privacy if generated carefully. However, the absence of a universally accepted definition for synthetic data complicates regulatory efforts, making it crucial to establish clear terminology in the rapidly evolving landscape of data usage and privacy. As an example, the United States (US) Census Bureau presents synthetic data in computer programming as entirely simulated data constructs for testing, free from real‐world constraints. At the same time, they recognize that in statistics, synthetic data often depict the amalgamation of multiple sources to produce detailed estimates [2].

We define synthetic data in line with the glossary of US Food and Drug Administration (FDA) on Digital Health and Artificial Intelligence [3] as, quoting:

Data that have been created artificially (e.g., through statistical modeling, computer simulation) so that new values and/or data elements are generated. Generally, synthetic data are intended to represent the structure, properties, and relationships seen in actual patient data, except that they do not contain any real or specific information about individuals.

Importantly, we define observed (or ‘true’) data as data that are obtained by direct measurement of collection from real‐world events (hence including Randomized Controlled Trials (RCT) and Real‐World Data (RWD)), and that are typically used as input to produce synthetic data [4].

In line with a recent discussion on the topic by Selvarajoo & Maurer‐Stroh [5], for a clearer distinction, we also pose that synthetic data can be broadly categorized into process‐driven and data‐driven approaches:

- Process‐driven synthetic data are generated using computational or mechanistic models based on biological or clinical processes and has been an established and regulatory‐accepted paradigm for decades [6, 7, 8]. These models typically use known mathematical equations (e.g., ordinary differential equations (ODEs)), such as pharmacokinetic (PK) and pharmacodynamic (PD) models and agent‐based simulations [9, 10]. The models are first developed to explain an observed behavior and are then subsequently used to generate simulated or synthetic data using the same model for different conditions or situations [11].
- Data‐driven synthetic data rely on statistical modeling and machine learning (ML) techniques, including sequential ensembles of decision trees, Variational Autoencoders (VAEs), and Generative Adversarial Networks (GANs), that have been trained on actual (“observed”) data and create synthetic datasets that preserve population‐level statistical distributions.

Nevertheless, there is still no generally accepted terminology when it comes to “synthetic” data, particularly when referring to data constituting external control arms (ECAs) in the context of clinical trials and drug development. According to the FDA draft guidance in 2023 [12], the ECA is defined as “..a group of people, treated or untreated, from an earlier time (historical control), …, or during the same period (concurrent control) but in another setting.” It’s worth noting that this draft guidance does not currently address whether an ECA can be composed of artificial or synthetic data; indeed, “synthetic control arms (SCAs)” or “virtual control arms (VCAs)” have also been used, we might argue “incorrectly” in light of the above definitions provided, as synonyms of ECAs [13, 14]. As an example, and as will be showcased in the upcoming sections of this manuscript, the ECA approach has been indicated with mixed nomenclatures when harnessing observed data for external controls from sources such as electronic health records (EHRs), administrative claims, patient‐generated information, disease registries, and prior clinical trial data [15], all with the final aim to offer an alternative to internal control groups [16].

### 1.1 RWD For ECAs: An Established Paradigm?

Despite the option to seek a (randomized) concurrent control still representing the gold standard in drug development, ECAs obtained from RCT or RWD sources have gained substantial traction particularly in contexts of providing supportive evidence (rather than confirmatory), with numerous drugs (particularly for rare diseases and unmet medical needs) approved to market through this approach [17], exemplifying the utility of such (observed) data from actual patient experiences. These ECAs serve as a critical component in observational studies and for comparative analyses where RCTs may be infeasible or unethical [18]. Indeed, from a regulatory standpoint, the FDA's publication of guidelines specific to the use of external data sources in constructing ECAs for drug development constituted another step towards the acceptance of RWD and experimental cohorts role in this approach [12]. As for the European context, the study by Wang et al. [19] provided a focused review, indicating that 18 European Medicines Agency (EMA)‐approved oncology drugs incorporated 24 ECAs (defined within that manuscript as “data derived outside the concurrent clinical trial”) obtained from external data sources from 2016 to 2021. Despite this progress, the authors also identified critical hurdles that such data face in the context of EMA evaluations: about one‐third of the ECAs were not considered supportive by the EMA, often due to issues related to lack of patient population heterogeneity and gaps in outcome assessments within the external data sources.

### 1.2 AI‐Generated Data: New Frontier With New Complexities

In the context of data‐driven (hence not process‐driven) generation processes [5], generated synthetic data (also referenced as “artificial” [20] or “simulated” data [21, 22]) have been in use for many years, albeit to a lesser extent than today. An example is imputation, i.e., filling in missing values: Artificial Intelligence (AI) models allow users to go beyond simpler heuristics such as using the mean (for numerical) or mode (for categorical) value by learning how other features influence the value to be filled in. Dong et al. provide an example of this as an application in healthcare [23].

However, it is only in the last decade that methodological advances in AI (e.g., the attention mechanism [24]) and sufficient computing power [25] have made it possible to reliably generate high‐fidelity synthetic values at the level of *entire datasets*. Another important enabling factor is *self‐supervised pre‐training*: a family of techniques to train an AI model to be generally useful on a variety of tasks by utilizing large amounts of data, without the need for human annotations [26, 27]. When it comes to clinical trials, pre‐training may enable synthetic data generation for ECAs, as data from control arms of different trials may be combined in a sufficiently large dataset to feed these algorithms [16].

Modern, data‐driven, generative AI models include GANs, VAEs, Diffusion Models (DMs), and Transformers [24, 28, 29, 30]. These models operate in two phases. Firstly, a model is trained using observed data: model parameters are adjusted such that the synthetic data produced by the model is similar to the original data. After the training, the model parameters are fixed, with the model now ready to generate synthetic data with statistical properties that are quasi‐identical to those of the original observed data source [31] without the generated data being directly linked to any particular individual present in the originating data [32]. In this second phase (inference), the AI model can be used to generate, or “sample,*”* synthetic data at will. For a more detailed review on the topic, we refer to [33].

With the advent of generative AI, the terminology of what constitutes “synthetic” data has been extended further, with some authors, we argue righteously in light of the rationale and definitions above, referring to “synthetic” as data created via generative AI techniques and/or process‐driven methods [5, 31], [34, 35, 36, 37]. Therefore, it has become important to distinguish between what was previously called “synthetic” (i.e., patient data possibly collected from myriad sources) and data that are generated (i.e., artificial data) by data‐ or process‐driven methods [5] to avoid confusion about the type of data being actually used [31].

### 1.3 Study Rationale

This paper endeavors to map, by means of critical literature review, the usage of the term “synthetic” with reference to data, delineating its provenance from observed (‘true’)‐derived constructs to generated data. We also reference *in silico* trial approaches that may involve the use of simulated data or virtual patient cohorts, framing these within more established process‐driven paradigms of synthetic data [5]. Finally, we analyze the evolving landscape of terminology in synthetic data research and propose a framework to mitigate ambiguity in its interpretation and application.

## 2 Methodology

This review employs a critical narrative approach to explore the understanding of terminology for different data sources in healthcare and drug development settings. Unlike systematic reviews that focus on answering specific, narrow questions through predefined methods, a critical narrative review allows for a broader examination of diverse studies, providing interpretation and critique across a wider scope of literature [38, 39].

### 2.1 Search Strategy

The literature search was conducted from 1986 to 2025 using the PubMed database to identify relevant studies. The following search query was employed to retrieve articles:

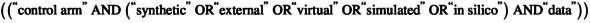




This query was designed to capture studies discussing control arms that utilize either synthetic or external data and other possible derivatives thereof. By specifying “control arm” and “data,” the search focused on relevant research involving these data types. The use of “synthetic” OR “external” OR “virtual” etc., broadened the scope to include various process‐driven methodologies spanning from more established contexts [40], ensuring a review of how these data types are referred to in medical and scientific research.

The search returned a total of 208 results, which were then screened in terms of content to make sure that the literature contained relevant information for the rationale of the manuscript. The final selected results were then summarized in Table S1 of this manuscript, accounting for *n* = 91 instances.

## 3 Discussion

### 3.1 The Multiplicity of “Synthetic Data”

The literature research revealed a bifurcation in the use of this term. On one side, synthetic data, in line with the data‐driven intuition proposed in Selvarajoo & Maurer‐Stroh [5], are referenced as generative AI outputs—artificial constructs devised through advanced computational models, such as GANs. These artificially produced datasets, also sometimes labeled as “false” [41] or “fake” data in different contexts [42, 43, 44], serve several purposes, primarily in exploratory and modeling capacities to simulate scenarios, patterns, or outcomes that may not be feasible or ethical to generate through traditional clinical trials. Pioneering studies by authors like Azizi et al., El Kabbaj et al., Fisher et al., and D'Amico et al. have contributed to this growing body of knowledge, pushing for the usage and recognition of this artificial data (defined as *synthetic data* in their works) as a possible proxy for observed (‘real’) data in different therapeutic areas [20, 45, 46, 47]. It is noteworthy that other recent works have also adopted the same notation [34, 35, 36, 37, 41, 48] and other authors like Alloza et al. have also sought to establish the role of artificial data (still referred to as *synthetic data*) in shaping regulatory decision‐making processes [31].

Interestingly, a narrative review by Gonzales, Guruswamy, and Smith also pointed out that the term “synthetic data” has been widely used to characterize datasets in various synthesized forms and levels [49]. They also describe three broad categories of synthetic data, specifically: (i) *Fully Synthetic Data*: Data that are completely artificial and do not contain any real data; (ii) *Partially Synthetic Data*: Describes datasets where only certain sensitive variables are replaced with synthetic counterparts, hence maintaining some level of real data; and (iii) *Hybrid Synthetic Data*: Data created by combining both real and synthetic data [49]. While partially synthetic data modify only selected attributes within real datasets (hence risk for reidentification is still present), hybrid synthetic data blend entire synthetic records with real records, offering strong privacy protection while maintaining high utility compared to the first two categories.

On the other hand, based on our findings, the term RWD was generally understood as referring to authentic (“observed”) patient data, but has been also used in combination with the term “synthetic”, particularly in the context of clinical trials (e.g., single‐arm trials). Indeed, Boyne et al. and Van Le et al. used the term “Real‐World Synthetic Control Arm” to describe RWD (obtained respectively from a national cancer registry and various clinical sites/research database sources) when used to construct comparative analyses in the absence of traditional randomized control arms [50, 51]. Similarly, Popat et al., along with Banerjee et al., Burcu et al., O'Haire et al., Neehal et al., Thorlund et al., Yoshino et al., and Zhu et al., opted for the designation “Synthetic Control Arm” or “Synthetic Controls” [52], eschewing references to observed data with such nomenclature [53, 54, 55, 56, 57, 58, 59, 60]. Interestingly, the work by Burcu et al. [53], also endorsed by the International Society for Pharmacoepidemiology (ISPE), posed the perspective that “External control arms are also called ‘synthetic’ control arms as they are not part of the original concurrent patient sample”. While this definition provides an endorsed framework within the context of external controls, we believe it does not fully encompass the broader scope introduced by recent AI‐driven methodologies for generating synthetic data. As these innovations continue to redefine the landscape of data generation in clinical research, a shared discussion with scientific societies becomes increasingly relevant to refine definitions and ensure alignment with emerging technological and regulatory perspectives. Meanwhile, in their work Uemura et al., used the term “External Synthetic Control” to refer to RWD whereas Serrano et al. pose that “External control arms include patient‐level real‐world data, prospective cohorts or registries, and *synthetic control arms* elaborated from pooled or individual clinical trial data”, adding another layer of complexity (and, potentially, confusion) to the matter [13, 61]. Menefee et al. and Walker et al. [62, 63] define data from previously conducted randomized trials (hence true/observed [64]) as “Synthetic Control Arms”, whereas Davi et al. define SCAs an “external control constructed from patient‐level data from previous clinical trials to match the baseline characteristics of the patients in an investigational group and can augment a single‐arm trial” [65, 66].

Another perspective, possibly in contrast with the concept of “*hybrid*” previously elucidated by Gonzales, Guruswamy, and Smith [49], was noted in Li et al. [67] defining the mixture of RWD and clinical trial data as a “hybrid control arm”, similarly to Tan et al., Sengupta et al., Zou et al., and Neehal et al. [58, 67, 68, 69, 70], but clashing with the definition by Kurki et al. [15], wherein the combination of RWD with RCT data is simply defined as an ECA.

In contrast to the previous mixed scenario of definitions, similarly to the above mentioned work by Kurki et al. [15], the majority of papers included in this critical review simply referred to a observed (RWD or RCTs) data‐composed control arm as an “ECA”, hence possibly pointing toward an emerging consensus over the usage of such terminology [71, 72, 73, 74, 75, 76, 77, 78, 79, 80]; please see Table S1 for a complete list of references.

Keeping in mind once again the proposed distinction between synthetic data as generated by a data‐driven vs. a process‐driven methodology [5], the discourse on data in clinical research acquires an additional layer of complexity with the introduction of the term “virtual controls” as in Switchenko et al. and Strayhorn [14, 81]. “Virtual controls,” as per Strayhorn and Switchenko et al., involve the use of actual observed outcome data from untreated individuals, coupled with statistical techniques to create counterfactual scenarios, thus offering a comparative baseline without the ethical concerns of withholding treatment. In another description, the “virtual control arms” nomenclature was utilized to refer to a deep learning (hence data‐driven) algorithm trained on data from historical control patients and able to generate a likely outcome in the form of biomarker status or a clinical endpoint [82]. In another work, Chen et al. defined virtual control arm as generated by bootstrapping observed data with replacement, whereas Nicholson et al. adopted such nomenclature when referring to data generated by a machine learning prediction algorithm [83, 84], hence referring to data‐driven methods for synthetic data generation [5]. Differing from the previous work, and pointing towards process‐driven methods for synthetic data generation [5], Folse et al. and Visentin et al. exploited the terminology of *virtual patients* as data generated by an ODEs model representing the physiological and disease pathways in cardiovascular events [85] and model generated data, respectively [86], whereas Dutta et al. adopted the “simulated historical control” when referring to data obtained via bootstrap of a Phase III trial data [87]. In another work, the ECA was referred to in a more generic way as “patients collected from data sources external to the single‐arm trial,” with synthetic data referred to as data obtained by means of “synthetic simulations” [88]. Ultimately, Suissa utilized “simulated data” to refer to data generated via exponential, and survival outcome distributions, whereas McMahon et al. adopted the terminology of “simulated study arm” to refer to study arms composed of simulated patients generated by a state‐transition model analyzed as patient‐level Monte‐Carlo simulation [89, 90].

To conclude, in their work also co‐authored by an FDA member, Seeger et al., in contrast with ISPE's endorsed perspective by Burcu et al. [53], addressing that “synthetic controls is sometimes used interchangeably with external control groups”, articulated that the dual use of “synthetic controls” when referring to observed (‘true’) data can lead to ambiguity, especially with the implication that the data might be “partially fabricated” [16]. To mitigate this confusion and ensure clarity (“*Due to the potential for confusion across these uses*…”), the term “external control group” was preferred by Seeger et al. when describing observed data that serve as a benchmark or point of reference in observational studies or clinical trials. The same nomenclature of “external control groups” or “arm” was preferred in other works [19, 91, 92] (see Table S1 for a complete reference list), as well as in a recent systematic review on the use of ECAs in immune‐mediated inflammatory diseases [93]. Acknowledging the lexical overlap between “external control,” “historical control,” and “synthetic control” observed in the literature, Wang et al., in line with the International Conference on Harmonization (ICH) definition (2000), align with the view that “external control” should be the term of choice for controls derived externally to the current clinical trial, to avoid the misconceptions that may arise from using other terms [19, 94].

### 3.2 Emerging Definitions for Synthetic Data and RWD in EU, US, and UK Legal and Regulatory Frameworks

From the legal and regulatory framework, definitions have also emerged, both for synthetic data and RWD (the latter intended as a subset of observed/true data). The EU Data Governance Act (DGA) is the only legal text referring to synthetic data described as a “privacy‐preserving method that could contribute to a more privacy‐friendly processing of data” [95]. In line with our views, EU Policy texts are also referring to synthetic data (European Commission, 2024) along with data protection‐specific sources, as “artificial data that is generated from original data and a model that is trained to reproduce the characteristics and structure of the original data” [96]. As part of the latest developments from the EU AI Act, synthetic data will be associated with so‐called “general‐purpose AI models” and there will be specific requirements in terms of risks and methodology (for generative AI systems and models) [97].

The EMA's, draft reflection paper on the use of AI in the medicinal product lifecycle mentions synthetic data as an instrument to “deploy differential privacy techniques” and for “increasing model performance” [98]. While the Medicines and Healthcare products Regulatory Agency (MHRA) does not have a formal position paper on the matter, a commentary authored by MHRA defined synthetic data as, “artificial data that mimic the properties of and relationships in real data” [99].

RWD are defined by EU legal texts as “health data generated outside of clinical studies” [100], a broad definition potentially encompassing both synthetic and non‐synthetic data. The EMA and MHRA similarly define RWD as data relating to patient health status or delivery of health care collected outside of a clinical study/in routine clinical practice [101, 102]. The FDA defined RWD as “data relating to patient health status and/or the delivery of health care routinely collected from a variety of sources” [103, 104], while also providing in a recent commentary an useful distinction (in the context of true‐observed data) between primary data collection and secondary data analysis, stressing the need for clarity in terminology of study designs [105].

With regards to synthetic data intended as artificially (hence as a product of a data‐driven method [5]) generated, the regulatory landscape is adapting to this technology. As already stressed earlier, there is growing consideration for integrating novel methodologies like AI‐generated synthetic data into the evidence generated to possibly support regulatory decision‐making of medical products [48, 98, 106]. The MHRA and its Clinical Practice Research Datalink (CPRD) has been leading research efforts on synthetic data generation, including applications of high‐fidelity synthetic data for purposes like validation of AI algorithms, data augmentation in the context of clinical trials for boosting sample sizes, and conditional boosting to address biases due to underrepresentation [107, 108].

The EMA has shown a keen interest in the potential of AI, as shown in their reflection paper on AI in the medicinal product lifecycle, which acknowledges the significance of data augmentation techniques such as synthetic data in expanding training datasets for AI algorithms [98], while also stressing concepts such as generalizability and fairness of the models utilized/developed.

With regards to the US, the FDA has already started to recognize generative AI's potential, reporting the authorization of 1016 AI/ML‐enabled medical devices as of December 2024 [109]. In the pharmaceutical realm, draft guidance on the use of AI in the drug development process have recently been published [106, 110], however, no specific recommendations are outlined for the use of synthetic data or generative AI models in particular. Collectively, the regulatory agencies appear to be at various stages of recognizing and incorporating AI‐generated (hence data‐driven based [5]) synthetic data into their methodologies. All agencies appear to concur on the potential of synthetic data to enhance model performance and contribute to the medicinal product lifecycle, yet no drug or medical device has been registered using solely or predominantly synthetic data (as artificially generated data from a data‐driven model) e.g., as a comparator arm [31]. It is reasonable to expect that quality aspects concerning synthetic data, possibly to be factored within statistical analyses, will need to be accounted for [111]. In fact, for the special case of predicting future outcomes given patient's baseline features (a use case often referred to as “digital twins”), a special case of ANCOVA has been referenced in regulatory discussions [112].

Besides new regulations and guidelines, it remains crucial for adopters of synthetic data to adhere to best practices and guidance documents related to data privacy, cybersecurity, and software validation in general. Moreover, given the current lack of an extensive guideline on the topic, the principles from the FDA's guidance's on the Use of Real‐World Evidence may be applied to synthetic data, particularly in terms of ensuring data quality and reliability [103, 104, 106, 109, 113]. The underlying principles about software quality from the FDA's guidance on Computer Software Assurance for Production and Quality System Software can also be relevant to the algorithms and processes used to generate synthetic data [114], an approach that mitigates risks while also positioning organizations to adapt swiftly to new regulations as they are drafted and implemented.

In this context, following the recent issue of Good Machine Learning Practice for Medical Development^1^ principles by FDA, MHRA and Health Canada (a source of information deemed general enough to also guide the application of AI/ML‐methods in biopharmaceutical development [115] given the current absence of official comprehensive guidances) a first attempt to define some best practice for the development, evaluation and use of *in silico* methodologies—which, to varying extents, may involve the use or generation of synthetic data via data or process driven methods—is represented by the position report, “Toward Good Simulation Practice: Best Practices for the Use of Computational Modelling and Simulation in the Regulatory Process of Biomedical Products” [116]. The consensus process involved experts worldwide working in academia, healthcare, industry, and regulatory bodies, including a team of 13 FDA computer modeling and simulation (CM&S) experts covering all three medical product centers: Center for Devices and Radiological Health (CDRH), Center for Drug Evaluation and Research (CDER), and Center for Biologics Evaluation and Research (CBER). Notably, the authors highlight in their report that current regulatory frameworks for assessing in silico methodologies do not align neatly with the traditional distinction between medicinal products and medical devices. According to the authors, these methodologies necessitate both elements of technical validation—typical of medical device regulatory pathways—and aspects of clinical validation, more commonly associated with medicinal product approvals. These initiatives are particularly interesting considering the key challenges associated with AI supported drug development endeavours highlighted by Nene et al. (2024) from the regulatory and sponsor perspectives [117]. On the regulatory side, difficulties such as inadequate description of data and insufficient evaluation or validation of models were emphasized. From the sponsor’s viewpoint, unclear model requirements and insufficient guidance on relevant cases of interest were identified as major concerns. Addressing these challenges will likely be critical in improving the regulatory acceptance and practical implementation of innovative methodologies. Beyond the regulatory framework, it is also essential to consider the existing legal frameworks upon which the different authorities act. In the EU, for example, existing medical device and in vitro diagnostic regulations (MDR/IVDR) and pharmaceutical laws do not explicitly prohibit the use of synthetic data as a supporting element of clinical evidence [118, 119]. For medical devices, the MDR per se does not prohibit using evidence generated through CM&S [120, 121], and therefore (artificially generated in terms of process‐driven method [5]) synthetic data. For medicinal products, it is noteworthy that the EU pharmaceutical reform package even refers to “considering new approach methodologies in place of animal testing,” including “in silico tools” [122], but in light of the related distinctions between synthetic data as generated by a more innovative data‐driven (e.g., AI and related models) approach rather than an established process‐driven one (e.g., CM&S as in QSP), more details and case examples will be required to determine their regulatory acceptance and differences.

In the presence of no explicit legal prohibitions, it is important that regulatory agencies and competent bodies take the initiative to align on these definitions and provide guidance on the use of synthetic data for generating evidence for medical products—so that healthcare stakeholders do not operate in a legal vacuum.

## 4 Addressing Emerging Issues With Synthetic Data

As the application of synthetic data in healthcare and drug development continues to grow, several critical issues need to be addressed to ensure the data's reliability, provenance, and transparency [117].

### 4.1 Provenance of Synthetic Data

Provenance, referring to the origin and history of data, provides a detailed record of its creation, transformation, and usage [123]. In the context of synthetic (as artificially generated) data, especially for the purpose of data augmentation, establishing robust provenance mechanisms is essential to maintain trust and credibility [102]. Unlike observed data, which usually has a clearer origin, synthetic data can be generated from algorithms and models that may combine multiple data sources [124] or mathematical models of an underlying biochemical process [5]. This complexity, as also proposed by The Data & Trust Alliance, necessitates detailed documentation of the models used, the observed (‘real’) data inputs (for data‐driven generation processes), and the synthetic data generation methodology [125].

To tackle these challenges, developing comprehensive metadata standards is crucial. These standards should document the data generation process, including the algorithms used, parameters set, and input data characteristics [116]. Storing this metadata alongside the synthetic data provides context and supports reproducibility [126].

### 4.2 Distinguishing Synthetic and Observed Data

As synthetic data becomes more integrated with observed data sources [49], distinguishing between the two is crucial to avoid misinterpretations and ensure appropriate usage in clinical research. The potential for data mixing, where synthetic and real data are combined, might pose significant challenges. Researchers may face difficulties identifying synthetic data elements, leading to potential biases or errors in analysis. Ensuring transparency in data usage and analysis is paramount, particularly when synthetic data augments observed datasets.

Adopting clear labeling practices where synthetic data are explicitly tagged and possible to be separated from observed data can mitigate these challenges. This may be achievable through data flags or markers embedded within the datasets. Furthermore, providing detailed documentation and visual aids, such as data lineage charts, may delineate the proportions and sources of synthetic and real data within mixed datasets. These practices could potentially enhance clarity and reduce the risk of misinterpretation.

#### 4.2.1 Developing Data Cards for Transparency

To address transparency issues, the concept of data cards has emerged as a potential solution [127]. Data cards are structured summaries that provide critical information about datasets, including their provenance, composition, and intended use. These cards may include detailed information about the original sources of the data, including any observed data sources (e.g., RWD, RCTs or mixtures thereof) used as input for generating synthetic data. Additionally, a description of the synthetic data generation process, including the algorithms and models employed, parameter settings, and any preprocessing steps, may be included [125].

Data cards may also highlight key characteristics of synthetic data, such as distributions, correlations, and outliers, through statistical summaries and visualizations [127]. Providing clear guidelines on the appropriate use of the data, potential limitations, and any known biases or uncertainties can strengthen the utility value of the data cards. Developing standardized templates for data cards that can be universally applied across different data and research projects will help ensure consistency. Leveraging automated tools to generate data cards as part of the synthetic data creation workflow can further enhance transparency and reduce manual effort.

### 4.3 Data Synthesis and Replicability via Generative AI

The data‐driven AI models that are utilized to produce synthetic data, while powerful, are imperfect and can fail to achieve their goals of producing high‐quality synthetic data in a variety of ways [128], some of which we find important to highlight.

Firstly, synthetic data that are generated by necessity inherits the properties of the observed data distribution used to train an AI model, e.g., if only data from a specific demographic are shown to the model, the model will not produce data relevant to other demographics. This makes it important to carefully consider which data are used to train the model and whether it is consistent with the intended use of the synthetic data. Secondly, even if the original data contain all relevant demographics, they may not be reproduced by generative AI, as it may struggle to represent less frequent data [129]. Ensuring careful testing of synthetic data is vital to detect and address such issues [129]. Finally, a generative AI model may learn the data *too well*, i.e., it may memorize some real data points and output them under the guise of “synthetic” data [130]. This may endanger patient privacy and break relevant laws such as the General Data Protection Regulation (GDPR). Measuring privacy risks is an open research topic with no well‐established procedures that nonetheless should not be ignored when applying generative AI in practice.

In terms of data replicability, El Emam et al. emphasize that the replicability of analyses performed on synthetic (as artificially generated) health data is a crucial factor in determining its validity for research or decision‐making use [111]. Their study demonstrates that for synthetic data to yield reliable results, at least 10 datasets of the same size as the original should be generated and analyzed using multiple imputation combining rules. Moreover, the study highlights the superiority of sequential synthesis (a generative approach used to construct synthetic datasets by iteratively modeling each variable conditional on the previously synthesized ones, ensuring that dependencies among variables are preserved in the synthetic dataset) over GANs in replicating real‐world analysis outcomes, ensuring high decision agreement, low bias, and appropriate confidence interval coverage.

Overall, a crucial aspect of evaluating the utility of synthetic data lies in its ability to yield conclusions that align with those derived from its original observed data source. If analyses conducted on synthetic and actual data lead to fundamentally different conclusions, the synthetic dataset may lack validity for decision‐making. Therefore, before releasing synthetic data for broader use, it is essential to assess its reliability through rigorous validation processes. This includes hypothesis testing and statistical analyses to ensure that key inferences—such as treatment effects or risk associations—remain consistent across synthetic and actual datasets. For such purpose, incorporation of model card, intended as a structured report detailing the technical characteristics of an AI model, benchmark evaluation results, and the context in which the model is designed to be used/methods employed to assess its performance [131] in such validation steps would enhance confidence in synthetic data as a viable tool for research and possibly regulatory decision‐making.

## 5 Conclusions

**FIGURE 1:**
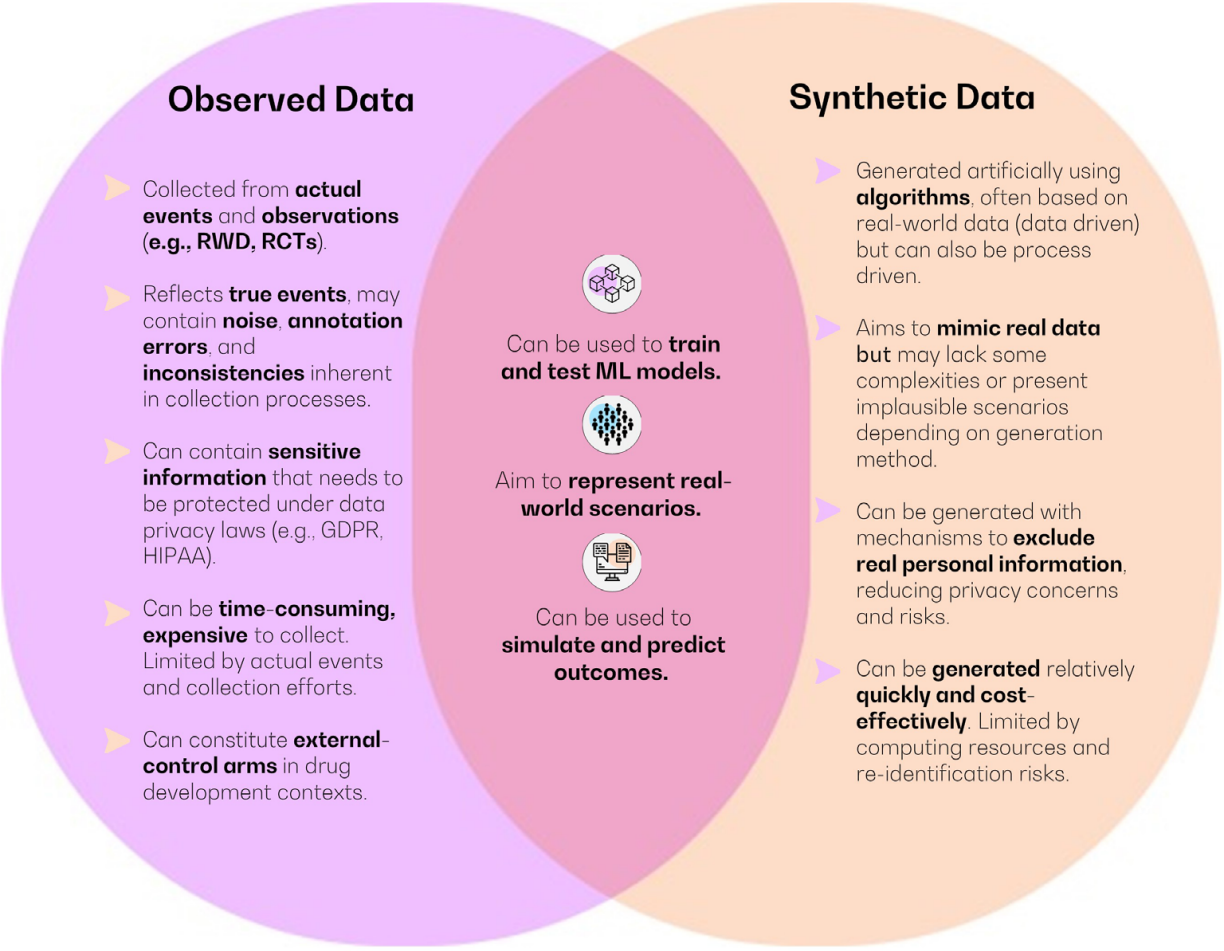
Comparison of observed (“true”) data and synthetic data via Venn diagram, illustrating the key characteristics, benefits, and limitations of each data type while highlighting their unique attributes and areas of overlap.

As the discourse on data provenance and classification unfolds within the clinical research and regulatory spheres, there is an undeniable surge in the dialogue surrounding its two main identities found in literature: data derived from observed data sources (e.g., RWD or RCTs), and that generated by AI and other statistical (e.g., data‐driven) means (artificial data), often and more frequently referred to as synthetic data [3, 5, 16, 49]. As depicted in Figure 1, observed data and synthetic data possess distinct characteristics and potential applications, with certain overlapping benefits that enhance their utility in healthcare research and, potentially, drug development.

While there is an established regulatory basis for the former, evidenced by the integration of RWD or historical RCTs data into ECAs for upwards of 45 licensed drugs [17], the latter is still navigating its regulatory definition. As for data deriving from in silico and CM&S (the *process‐driven* generated synthetic data [5]), the FDA, EMA, and MHRA routinely accept in silico evidence from physiologically based pharmacokinetic (PBPK), population pharmacokinetic (popPK) and/or pharmacodynamic (popPKPD), and exposure‐response (ER) modeling in drug development [113, 132, 133]. Notwithstanding, the emergent generation of synthetic data through AI (the *data‐driven* generated synthetic data [5]), as referenced in recent exploratory studies and regulatory endeavours, has yet to be defined in a harmonized fashion within regulatory frameworks, despite acknowledging the substantial potential of AI in this domain [48, 98]. This dichotomy—between data rooted in actuality and data “born” from algorithms—highlights a need for clarity and consensus from stakeholders.

There is a compelling imperative for the health care and drug‐development communities to define and distinguish these types of data and related derivations (see discussion on comparator arms in clinical trials and drug development in above sections) rigorously. The lack of clear distinction might dampen the current understanding and potential applications of these powerful tools, hence hampering the progress of possibly adopting AI‐generated data within regulated drug development pathways. To aid in clarifying this terminology, we suggest (Table 1) the following conventions: We define observed data as (‘real’ or ‘true’) data that are obtained by direct measurement or collection from real‐world events, (hence including RCT and RWD). We then define the term “real‐world data” as defined by the FDA [134] as data relating to patient health status and/or the delivery of health care routinely collected from a variety of sources. Examples of RWD include data derived from EHRs, claims and billing data, data from product and disease registries, patient‐generated data including in home‐use settings, and data gathered from other sources that can inform on health status, such as mobile devices. RWD sources (e.g., registries, collections of EHRs, and administrative and healthcare claims databases) can be used as data collection and analysis infrastructure to support randomized controlled trials, including acting as an ECA source.

**TABLE 1:** Terminology clarification and suggested definitions for conventions.

Term	Definition	Context/References
Observed data	Data that are obtained by direct measurement or collection from real‐world events, are typically used as input to produce synthetic data. Includes RCT and RWD	Current paper and necessity to distinguish observed data from synthetic (as artificial) data [96]
Real‐world data	Data relating to patient health status and/or the delivery of health care routinely collected from a variety of sources. Can be further categorized into primary data (collected directly from study participants, either retrospectively or prospectively) and secondary data (obtained from existing healthcare data collection infrastructures, such as administrative claims databases, EHR databases, patient registries, or individual patient medical records)	FDA definition [134], and [64, 105, 135] for primary and secondary RWD distinction
Synthetic data (or fully synthetic data)	Data that have been created artificially (e.g., through statistical modeling, computer simulation) so that new values and/or data elements are generated	Current paper, FDA definition, and scientific discussions on the topic [3, 5, 49, 96]
Partially synthetic data	Data in which only selected variables are replaced with a synthetic (i.e., artificial) generated data values	Current paper, and as posed by authors from the US Department of Health and Human Services and Department of Health Administration and Policy [49]
Hybrid synthetic data	Data integrating observed (‘real’ or ‘actual’) and synthetic data	
External control arm	Control or comparator groups in clinical studies derived from external (concurrent) or historical sources of data. Depending on the nature of data used, it should be classified as a synthetic control arm if fully synthetic data are used, a partially synthetic control arm if only selected variables are replaced with synthetic data, or a hybrid synthetic control arm if records of observed and synthetic data are integrated	Current paper and as posed in regulatory contexts [12, 16, 17]; if synthetic data are included in an ECA, it should be explicitly reported whether the data generation method was process‐driven or data‐driven
Process‐driven synthetic data	Data that have been created artificially using mechanistic or computational models that simulate biological/clinical processes	Used primarily in bioinformatics, computational biology, and clinical pharmacology (e.g., PBPK and popPKPD modeling) for simulating biological systems, widely recognized, regulated, and accepted by regulatory bodies [5]
Data‐driven synthetic data	Data that have been created artificially after models have been trained on actual true/observed data	Typically derived from AI models trained on (true/observed) real‐world or controlled datasets, increasingly employed for dataset augmentation, validation, or analytical purposes, yet currently less established within regulatory frameworks [5]

The term “synthetic” in the context of data is reserved for artificial data that are generated via algorithmic processes, as recently suggested in a regulatory context [3]. It is important to distinguish between synthetic data derived from process‐driven and data‐driven methods [5], as they rely on different underlying assumptions, technical frameworks, and regulatory considerations. *Process‐driven* synthetic data, such as those generated through mechanistic modeling (e.g., QSP), have long been established and widely accepted in drug development, whereas *data‐driven* synthetic data, often produced using AI and ML, remain relatively novel with no (to the best of our knowledge) regulatory precedent in the context of drug development/drug approval. The classification of synthetic data provided by Gonzales, Guruswamy and Smith [49] into fully, partially, or hybrid synthetic data is also useful to distinguish between subtypes of synthetic data. In the context of clinical trials using control or comparator arms drawn from RWD or historical controls from previous clinical trials, we suggest, in line with Seeger et al. [16], the use of the term “External Control Arm (ECA)” with specification of the source of the external controls (i.e., by means of observed data sources, generative AI techniques, or possible mixtures thereof).

In conclusion, it is evident that a collaborative dialogue among various communities, including academia, clinicians, industry, and regulatory advisors, can foster a shared understanding and guide the thoughtful exploration of synthetic data's potential in its finest declinations. It is through collective insights and expert discussions that the path forward can be envisioned, encouraging a harmonized perspective on synthetic data's role in advancing medical science and drug development.

## Conflicts of Interest

G.P., V.C., M.T., and D.R. are employees of InSilicoTrials Technologies S.p.A.; M.V. and A.C. are employees of InSilicoTrials Technologies B.V., two companies operating in modeling and simulation for drug development purposes. L.E. is the chief executive officer of InSilicoTrials Technologies S.p.A. A.V. is an equity‐holding employee of Unlearn.AI Inc., a company that creates digital twin generators to forecast patient outcomes. C.F. is the chief executive officer of Unlearn.AI Inc. S.D. is the chief executive officer and chief technology officer of Train S.r.l., a company involved in the development of digital twin technology and synthetic data generation for precision medicine and drug development. The other authors declared no competing interests for this work.

## Supporting information


Table S1.

## Endnotes

^1^

https://www.fda.gov/medical‐devices/software‐medical‐device‐samd/good‐machine‐learning‐practice‐medical‐device‐development‐guiding‐principles.
