# Supplementary material for: Synthetic Data in Healthcare and Drug Development: Definitions, Regulatory Frameworks, Issues
Source: CPT Pharmacometrics Syst Pharmacol. 2025 Apr 7;14(5):840–52. doi: 10.1002/psp4.70021 (PMC12072219; doi:10.1002/psp4.70021)
Supplement: Supplementary file 1 — Table S1. [file PSP4-14-840-s001.docx]

**Appendix**

**Table S1** Varying meanings assigned to ‘synthetic data’ and its declinations.

| Reference | Synthetic Data Declination |
| --- | --- |
| (Abbas et al., 2022) | Historical Control Data defined as obtained from randomized phase III Trial |
| (Abé et al., 2024) | Real-world data as External Control Arm |
| (Acorn AI, 2020) | Synthetic data defined as a type of External Control that is generated using patient-level data from patients external to the trial with the goal of improving the interpretation of uncontrolled trials, which can enable better product development decisions |
| (Alloza et al., 2023) | Generative AI data referred to as Synthetic Data |
| (Arora & Arora, 2023) | Generative AI data referred to as Synthetic Data, Real-world data referred to as Real Data |
| (Azizi et al., 2021, 2023) | Artificially Generated data referred to as Synthetic Data |
| (Backenroth, 2021) | Real-world data as External Control Arm. |
| (Banerjee et al., 2022) | Observational data referred to as Synthetic Control Arm |
| (Børø et al., 2023) | Real-world data as External Comparator/Control Arm. |
| (Boyne et al., 2023) | Population-level Real-World data referred to as Real-World Synthetic Control Arm |
| (Bruhn et al., 2017) | Nationwide administrative data referred to as Synthetic Controls |
| (Burcu et al., 2020) | Real-world data referred as external or Synthetic Control Arm |
| (Carrigan et al., 2020) | Real-world data referred as External Control Arm |
| (Carton et al., 2023) | Real-world data as External Control Arm. |
| (Chen et al., 2021) | Virtual control arm as generated by bootstrap with replacement. |
| (Curtis et al., 2022) | Real-world data as External Control Arm |
| (D’Amico et al., 2023) | Generative AI data referred as Synthetic Data |
| (Dang et al., 2023) | Real-world data as External Control Arm |
| (Davi et al., 2019, 2020) | Synthetic Control Arm is an External Control constructed from patient-level data from previous clinical trials to match the baseline characteristics of the patients in an investigational group and can augment a single-arm trial |
| (Davies et al., 2018) | Electronic health record (EHR) data referred to as external Real-world data |
| (Dutta et al., 2024) | Simulated Historical Control as obtained via bootstrap phase III trial |
| (El Kababji et al., 2023) | Generative AI data referred as Synthetic Data |
| (Farah et al., 2024) | Real-World Data referred to as External Control Arms |
| (Fisher et al., 2019) | Model generated data referred to as Synthetic Patients |
| (Folse et al., 2014) | Virtual patients as generated by an ODE model representing the physiological and disease pathways in cardiovascular events. |
| (Gonzales et al., 2023) | Synthetic Data distinguished in Fully Synthetic, Partially Synthetic, and Hybrid with different criterions |
| (Goulden et al., 2023) | Real-world data as External Control Arm |
| (Gray et al., 2024) | Real-world data as External Controls |
| (International Conference on Harmonization (ICH), 2000) | Guideline E10 defines an Externally Controlled Trial as “one in which the control group consists of subjects who are not part of the same randomized study as the group receiving the investigational agent, i.e., there is no concurrently Randomized Control Group” |
|  | External Control Arms (ECAs) may be categorized as Concurrent ECAs and Historical ECAs: Concurrent ECAs use subject data collected during the same time-periods as the subjects receiving the investigational agents, while historical ECAs use subject data collected at an earlier time |
| (Jacobs et al., 2023) | Generative AI data referred to as Synthetic Data |
| (Jaksa et al., 2022) | Real-world data as External Control Arm |
| (Jeon et al., 2023) | Real-world data as External Control Arm |
| (Jin et al., 2022) | Real-world data as External Control Arm |
| (Kang et al., 2023) | Generative AI data referred to as Synthetic Data |
| (Khachatryan et al., 2023) | Clinical trial data and Real-world Data as External Control Arm. Synthetic Control Arm as derived from model-based meta-analysis. |
| (Klein et al., 2024) | Real-world data as External Control Arm |
| (Kokosi et al., 2022) | Generative AI data referred to as Synthetic Data |
| (Kurki et al., 2024) | Real-world data combined with clinical trial data to define an External Control Arm |
| (H. Li et al., 2022) | Real-world data and Clinical Trial data to define Hybrid Control Arm |
| (J. Li et al., 2023) | Real-world data as External Control |
| (Loiseau et al., 2022) | Data external from single-arm trial as External Control Arm |
| (Martin et al., 2022) | Real-world data as Real-world Control Arm |
| (Mateos et al., 2023) | Clinical trial data as External Control Arm |
| (McMahon et al., 2008) | Simulated study arm as composed by simulated patients generated by a State-transition model analyzed as patient-level Monte-Carlo simulation. |
| (Mehtälä et al., 2023) | Real-world data as External Control Arm |
| (Menefee et al., 2019) | Synthetic Control Arms defined as Control Arms from previously conducted Clinical trials. |
| (Moreau et al., 2023, 2024) | Real-world data as External Control Arm |
| (Mishra-Kalyani et al., 2022) | Real-world data as External Control Arm |
| (Myles et al., 2023) | Artificial data that mimic the properties and relationships of real data are referred to as Synthetic Data |
| (Murray et al., 2021) | Clinical trial data as Historical Control data |
| (Narita et al., 2022) | Real-world data as External Control Arm |
| (Neehal et al., 2023) | Real-world data referred to as Synthetic Controls or Hybrid Control Arm when trial data is augmented with real-world data. |
| (Nicholson et al., 2018) | Virtual Control Arm as obtained by a ML prediction algorithm. |
| (Noguer et al., 2022) | Generative AI data referred to as Synthetic Data |
| (O’Haire et al., 2022) | Real-world data as Synthetic Control Arm |
| (Orbach et al., 2024) | Real-world data as External Control Arm |
| (Polito et al., 2024) | Real-world data as External Control Arm/Observational Comparator |
| (Polley et al., 2024) | Real-world data as External Control Data |
| (Popat et al., 2022) | Real-world data referred to as Synthetic Control Arm |
| (Rudrapatna et al., 2023) | Real-world data as External Control Arm |
| (Schneeweiss, 2024) | Real-world data as External Control Arm |
| (Schröder et al., 2021) | Real-world data as External Control Arm |
| (Seeger et al., 2020) | Detected Synthetic Control as used interchangeably with External Control groups, or when involves data generations for simulation studies that is based on actual data. |
| (Sengupta et al., 2023) | Hybrid control arm as mixture of Real-world and Clinical Trial data. |
| (Serrano et al., 2023) | Real-world data referred to as External and/or Synthetic Control Arm |
| (Silva et al., 2023) | Real-world data referred as External Control Arm/ Hybrid External Control Arms when collecting both retrospective and prospective data |
| (Siu et al., 2024) | External Controls defined as Control Arm Data derived outside the Clinical Trial |
| (Stanford University, 2023) | Model-based data (Deep Learning) referred to as Virtual Control Arm |
| (Strayhorn, 2021) | Real-world data referred to as Virtual Controls |
| (Struebing et al., 2024) | Real-world data as External Control Arm |
| (Suissa, 2021) | Simulated data as generated via exponential, salvage-related, and survival outcome distributions. |
| (Switchenko et al., 2019) | Real-world data referred to as Virtual Control Arm/Group |
| (Tan et al., 2022) | Real-world data referred to as Hybrid control arms (used when trial data and external RWD are mixed). |
| (Thorlund et al., 2020) | Real-world data referred to as Synthetic Control Data |
| (Thorlund et al., 2024) | Real-world data referred to as External Control Arm |
| (Uemura et al., 2023) | Real-world data referred to as External Synthetic Control Arm |
| (Umer & Adnan, 2024) | Generative AI data referred to as Synthetic Data |
| (Van Le et al., 2023) | Real-world data referred to as Synthetic Control Arm or External Control Arm |
| (Velummailum et al., 2023) | Real-world data as External Control Arm |
| (Visentin et al., 2014) | Model-generated data referred to as Virtual Patients |
| (Walker et al., 2024) | Synthetic Controls when data is sourced from prior trials or External Controls when sourced from Real-world data. |
| (Wang et al., 2023) | Real-world data as External Control Arm |
| (Wei et al., 2024) | Real-world data as External Control Data |
| (Yin et al., 2022, 2023) | Real-world data and Clinical trial data as External Control arm |
| (Yoshino et al., 2023) | Real-world data referred to as Synthetic Control Arm |
| (Zayadi et al., 2023) | Real-world data referred to as External Controls |
| (Zhou & Ji, 2021) | Simulated External Control data to refer to data generated by means of Bayesian additive regression trees. |
| (Zhu & Tang, 2022) | Real-world data referred as Synthetic Control Arm |
| (Zou et al., 2024) | Real-world data as external control arm, synthetic control arm also mentioned (Medidata). Hybrid control arm when trial data is augmented with external control arm. |

# **References**

Abbas, R., Wason, J., Michiels, S., & Le Teuff, G. (2022). A two‐stage drop‐the‐losers design for time‐to‐event outcome using a historical control arm. *Pharmaceutical Statistics*, *21*(1), 268–288. https://doi.org/10.1002/pst.2168

Abé, C., Keto, J., Lilja, M., Konradsen, M., Mesterton, J., Höglund, M., Lazarevic, V., Lehmann, S., & Juliusson, G. (2024). Cytarabine dose intensification improves survival in older patients with secondary/high-risk acute myeloid leukemia in matched real-world versus clinical trial data. *Leukemia & Lymphoma*, 1–9. https://doi.org/10.1080/10428194.2024.2363430

Acorn AI, a M. C. (2020). *Synthetic Control Arm in Clinical Trials*. https://www.medidata.com/wp-content/uploads/2020/11/Acorn-AI-Synthetic-Control-Arm-in-Clinical-Trials.pdf

Alloza, C., Knox, B., Raad, H., Aguilà, M., Coakley, C., Mohrova, Z., Boin, É., Bénard, M., Davies, J., Jacquot, E., Lecomte, C., Fabre, A., & Batech, M. (2023). A Case for Synthetic Data in Regulatory Decision‐Making in Europe. *Clinical Pharmacology & Therapeutics*, *114*(4), 795–801. https://doi.org/10.1002/cpt.3001

Arora, A., & Arora, A. (2023). Machine learning models trained on synthetic datasets of multiple sample sizes for the use of predicting blood pressure from clinical data in a national dataset. *PLOS ONE*, *18*(3), e0283094. https://doi.org/10.1371/journal.pone.0283094

Azizi, Z., Lindner, S., Shiba, Y., Raparelli, V., Norris, C. M., Kublickiene, K., Herrero, M. T., Kautzky-Willer, A., Klimek, P., Gisinger, T., Pilote, L., & El Emam, K. (2023). A comparison of synthetic data generation and federated analysis for enabling international evaluations of cardiovascular health. *Scientific Reports*, *13*(1), 11540. https://doi.org/10.1038/s41598-023-38457-3

Azizi, Z., Zheng, C., Mosquera, L., Pilote, L., & El Emam, K. (2021). Can synthetic data be a proxy for real clinical trial data? A validation study. *BMJ Open*, *11*(4), e043497. https://doi.org/10.1136/bmjopen-2020-043497

Backenroth, D. (2021). How to choose a time zero for patients in external control arms. *Pharmaceutical Statistics*, *20*(4), 783–792. https://doi.org/10.1002/pst.2107

Banerjee, R., Midha, S., Kelkar, A. H., Goodman, A., Prasad, V., & Mohyuddin, G. R. (2022). Synthetic control arms in studies of multiple myeloma and diffuse large B‐cell lymphoma. *British Journal of Haematology*, *196*(5), 1274–1277. https://doi.org/10.1111/bjh.17945

Børø, S., Thoresen, S., Boge Brant, S., & Helland, Å. (2023). Initial investigation of using Norwegian health data for the purpose of external comparator arms—An example for non-small cell lung cancer. *Acta Oncologica (Stockholm, Sweden)*, *62*(12), 1642–1648. https://doi.org/10.1080/0284186X.2023.2264484

Boyne, D. J., Dawe, D. E., Shakir, H., Joe-Uzuegbu, O., Farah, E., Pabani, A., Baratta, C., Brenner, D. R., & Cheung, W. Y. (2023). Comparative Effectiveness of Lurbinectedin for the Treatment of Relapsed Small Cell Lung Cancer in the Post-Platinum Setting: A Real-World Canadian Synthetic Control Arm Analysis. *Targeted Oncology*, *18*(5), 697–705. https://doi.org/10.1007/s11523-023-00995-1

Bruhn, C. A. W., Hetterich, S., Schuck-Paim, C., Kürüm, E., Taylor, R. J., Lustig, R., Shapiro, E. D., Warren, J. L., Simonsen, L., & Weinberger, D. M. (2017). Estimating the population-level impact of vaccines using synthetic controls. *Proceedings of the National Academy of Sciences*, *114*(7), 1524–1529. https://doi.org/10.1073/pnas.1612833114

Burcu, M., Dreyer, N. A., Franklin, J. M., Blum, M. D., Critchlow, C. W., Perfetto, E. M., & Zhou, W. (2020). Real‐world evidence to support regulatory decision‐making for medicines: Considerations for external control arms. *Pharmacoepidemiology and Drug Safety*, *29*(10), 1228–1235. https://doi.org/10.1002/pds.4975

Carrigan, G., Whipple, S., Capra, W. B., Taylor, M. D., Brown, J. S., Lu, M., Arnieri, B., Copping, R., & Rothman, K. J. (2020). Using Electronic Health Records to Derive Control Arms for Early Phase Single-Arm Lung Cancer Trials: Proof-of-Concept in Randomized Controlled Trials. *Clinical Pharmacology and Therapeutics*, *107*(2), 369–377. https://doi.org/10.1002/cpt.1586

Carton, M., Del Castillo, J. P., Colin, J.-B., Kurtinecz, M., Feuilly, M., Pierron, G., Arvis, P., Khadir, S. K., Sparber-Sauer, M., & Orbach, D. (2023). Larotrectinib versus historical standard of care in patients with infantile fibrosarcoma: Protocol of EPI-VITRAKVI. *Future Oncology (London, England)*, *19*(24), 1645–1653. https://doi.org/10.2217/fon-2023-0114

Chen, Z., Zhang, H., Guo, Y., George, T. J., Prosperi, M., Hogan, W. R., He, Z., Shenkman, E. A., Wang, F., & Bian, J. (2021). Exploring the feasibility of using real-world data from a large clinical data research network to simulate clinical trials of Alzheimer’s disease. *Npj Digital Medicine*, *4*(1), 84. https://doi.org/10.1038/s41746-021-00452-1

Curtis, J. R., Strand, V., Golombek, S., Zhang, L., Wong, A., Zielinski, M. C., Akmaev, V. R., Saleh, A., Asgarian, S., & Withers, J. B. (2022). Patient outcomes improve when a molecular signature test guides treatment decision-making in rheumatoid arthritis. *Expert Review of Molecular Diagnostics*, 1–10. https://doi.org/10.1080/14737159.2022.2140586

D’Amico, S., Dall’Olio, D., Sala, C., Dall’Olio, L., Sauta, E., Zampini, M., Asti, G., Lanino, L., Maggioni, G., Campagna, A., Ubezio, M., Russo, A., Bicchieri, M. E., Riva, E., Tentori, C. A., Travaglino, E., Morandini, P., Savevski, V., Santoro, A., … Della Porta, M. G. (2023). Synthetic Data Generation by Artificial Intelligence to Accelerate Research and Precision Medicine in Hematology. *JCO Clinical Cancer Informatics*, *7*, e2300021. https://doi.org/10.1200/CCI.23.00021

Dang, L. E., Fong, E., Tarp, J. M., Clemmensen, K. K. B., Ravn, H., Kvist, K., Buse, J. B., Van Der Laan, M., & Petersen, M. (2023). Case study of semaglutide and cardiovascular outcomes: An application of the C *ausal Roadmap* to a hybrid design for augmenting an RCT control arm with real-world data. *Journal of Clinical and Translational Science*, *7*(1), e231. https://doi.org/10.1017/cts.2023.656

Davi, R., Chandler, M., Elashoff, B., Ferris, A. S., Howland, A., Lee, D., Majumdar, A., Stewart, M., Strianese, L., Stuart, E., Yin, X., & Yver, A. (2019). Non-small cell lung cancer (NSCLC) case study examining whether results in a randomized control arm are replicated by a synthetic control arm (SCA). *Journal of Clinical Oncology*, *37*(15_suppl), 9108–9108. https://doi.org/10.1200/JCO.2019.37.15_suppl.9108

Davi, R., Yin, X., & Stewart, M. (2020). Exploring the validity of a synthetic control arm (SCA) for augmentation or replacement of a randomized control in difficult-to-study indications: A case study in relapsed or refractory multiple myeloma (R/R MM). *Journal of Clinical Oncology*, *38*(15_suppl), e20521–e20521. https://doi.org/10.1200/JCO.2020.38.15_suppl.e20521

Davies, J., Martinec, M., Delmar, P., Coudert, M., Bordogna, W., Golding, S., Martina, R., & Crane, G. (2018). Comparative effectiveness from a single-arm trial and real-world data: Alectinib versus ceritinib. *Journal of Comparative Effectiveness Research*, *7*(9), 855–865. https://doi.org/10.2217/cer-2018-0032

Dutta, R., Mohan, A., Buros‐Novik, J., Goldmacher, G., Akala, O. O., & Topp, B. (2024). A bootstrapping method to optimize go/no‐go decisions from single‐arm, signal‐finding studies in oncology. *CPT: Pharmacometrics & Systems Pharmacology*, psp4.13161. https://doi.org/10.1002/psp4.13161

El Kababji, S., Mitsakakis, N., Fang, X., Beltran-Bless, A.-A., Pond, G., Vandermeer, L., Radhakrishnan, D., Mosquera, L., Paterson, A., Shepherd, L., Chen, B., Barlow, W. E., Gralow, J., Savard, M.-F., Clemons, M., & El Emam, K. (2023). Evaluating the Utility and Privacy of Synthetic Breast Cancer Clinical Trial Data Sets. *JCO Clinical Cancer Informatics*, *7*, e2300116. https://doi.org/10.1200/CCI.23.00116

Farah, E., Kenney, M., Warkentin, M. T., Cheung, W. Y., & Brenner, D. R. (2024). Examining external control arms in oncology: A scoping review of applications to date. *Cancer Medicine*, *13*(13), e7447. https://doi.org/10.1002/cam4.7447

Fisher, C. K., Smith, A. M., Walsh, J. R., Coalition Against Major Diseases, Simon, A. J., Edgar, C., Jack, C. R., Holtzman, D., Russell, D., Hill, D., Grosset, D., Wood, F., Vanderstichele, H., Morris, J., Blennow, K., Marek, K., Shaw, L. M., Albert, M., Weiner, M., … Abbott, Alliance for Aging Research, Alzheimer’s Association, Alzheimer’s Foundation of America, AstraZeneca Pharmaceuticals LP, Bristol-Myers Squibb Company, Critical Path Institute, CHDI Foundation, Inc., Eli Lilly and Company, F. Hoffmann-La Roche Ltd, Forest Research Institute, Genentech, Inc., GlaxoSmithKline, Johnson & Johnson, National Health Council, Novartis Pharmaceuticals Corporation, Parkinson’s Action Network, Parkinson’s Disease Foundation, Pfizer, Inc., sanofi-aventis. Collaborating Organiza. (2019). Machine learning for comprehensive forecasting of Alzheimer’s Disease progression. *Scientific Reports*, *9*(1), 13622. https://doi.org/10.1038/s41598-019-49656-2

Folse, H., Sternhufvud, C., Andy Schuetz, C., Rengarajan, B., & Gandhi, S. (2014). Impact of Switching Treatment From Rosuvastatin to Atorvastatin on Rates of Cardiovascular Events. *Clinical Therapeutics*, *36*(1), 58–69. https://doi.org/10.1016/j.clinthera.2013.12.003

Gonzales, A., Guruswamy, G., & Smith, S. R. (2023). Synthetic data in health care: A narrative review. *PLOS Digital Health*, *2*(1), e0000082. https://doi.org/10.1371/journal.pdig.0000082

Goulden, S., Shen, Q., Coleman, R. L., Mathews, C., Hunger, M., Pahwa, A., & Schade, R. (2023). Outcomes for Dostarlimab and Real-World Treatments in Post-platinum Patients With Advanced/Recurrent Endometrial Cancer: The GARNET Trial Versus a US Electronic Health Record-Based External Control Arm. *Journal of Health Economics and Outcomes Research*, *10*(2), 53–61. https://doi.org/10.36469/001c.77484

Gray, C., Ralphs, E., Fox, M. P., Lash, T. L., Liu, G., Kou, T. D., Rivera, D. R., Bosco, J., Braun, K. V. N., Grimson, F., & Layton, D. (2024). Use of quantitative bias analysis to evaluate single-arm trials with real-world data external controls. *Pharmacoepidemiology and Drug Safety*, *33*(5), e5796. https://doi.org/10.1002/pds.5796

International Conference on Harmonization (ICH). (2000). *E10: Choice of Control Group and Related Issues in Clinical Trials*. https://database.ich.org/sites/default/files/E10_Guideline.pdf

Jacobs, F., D’Amico, S., Benvenuti, C., Gaudio, M., Saltalamacchia, G., Miggiano, C., De Sanctis, R., Della Porta, M. G., Santoro, A., & Zambelli, A. (2023). Opportunities and Challenges of Synthetic Data Generation in Oncology. *JCO Clinical Cancer Informatics*, *7*, e2300045. https://doi.org/10.1200/CCI.23.00045

Jaksa, A., Louder, A., Maksymiuk, C., Vondeling, G. T., Martin, L., Gatto, N., Richards, E., Yver, A., & Rosenlund, M. (2022). A Comparison of Seven Oncology External Control Arm Case Studies: Critiques From Regulatory and Health Technology Assessment Agencies. *Value in Health: The Journal of the International Society for Pharmacoeconomics and Outcomes Research*, *25*(12), 1967–1976. https://doi.org/10.1016/j.jval.2022.05.016

Jeon, J.-Y., Kim, M.-J., Im, Y.-J., Kim, E.-Y., Kim, J. S., Kwon, K. T., Hwang, J.-H., Kim, J. S., & Kim, M.-G. (2023). Development of an External Control Arm Using Electronic Health Record-Based Real-World Data to Evaluate the Efficacy of COVID-19 Treatment. *Clinical Pharmacology and Therapeutics*, *113*(6), 1274–1283. https://doi.org/10.1002/cpt.2882

Jin, Y., Li, J., Shen, L., Xu, J., Zhang, Y., Zhang, J., Pan, H., Qu, X., Chen, Y., Zhang, Q., Li, J., Sun, M., & Qin, S. (2022). A multi-center effectiveness comparison study of fruquintinib with constructed external control cohort of other targeted kinase inhibitors using real-world data in third-line treatment of metastatic colorectal cancer. *Frontiers in Oncology*, *12*, 1044328. https://doi.org/10.3389/fonc.2022.1044328

Kang, H. Y. J., Batbaatar, E., Choi, D.-W., Choi, K. S., Ko, M., & Ryu, K. S. (2023). Synthetic Tabular Data Based on Generative Adversarial Networks in Health Care: Generation and Validation Using the Divide-and-Conquer Strategy. *JMIR Medical Informatics*, *11*, e47859. https://doi.org/10.2196/47859

Khachatryan, A., Read, S. H., & Madison, T. (2023). External control arms for rare diseases: Building a body of supporting evidence. *Journal of Pharmacokinetics and Pharmacodynamics*, *50*(6), 501–506. https://doi.org/10.1007/s10928-023-09858-8

Klein, A., Toll, A., Stewart, D., & Fitzsimmons, W. E. (2024). Applying propensity methods to the United States transplant registry for external real-world evidence control arms for 5-year survival in the BENEFIT study. *American Journal of Transplantation: Official Journal of the American Society of Transplantation and the American Society of Transplant Surgeons*, *24*(2), 250–259. https://doi.org/10.1016/j.ajt.2023.09.019

Kokosi, T., De Stavola, B., Mitra, R., Frayling, L., Doherty, A., Dove, I., Sonnenberg, P., & Harron, K. (2022). An overview on synthetic administrative data for research. *International Journal of Population Data Science*, *7*(1). https://doi.org/10.23889/ijpds.v7i1.1727

Kurki, S., Halla-Aho, V., Haussmann, M., Lähdesmäki, H., Leinonen, J. V., & Koskinen, M. (2024). A comparative study of clinical trial and real-world data in patients with diabetic kidney disease. *Scientific Reports*, *14*(1), 1731. https://doi.org/10.1038/s41598-024-51938-3

Li, H., Tiwari, R., & Li, Q. H. (2022). Conditional borrowing external data to establish a hybrid control arm in randomized clinical trials. *Journal of Biopharmaceutical Statistics*, *32*(6), 954–968. https://doi.org/10.1080/10543406.2021.2021227

Li, J., Du, Y., Liu, H., & Yi, Y. (2023). An Improved Matching Practice for Augmenting a Randomized Clinical Trial with External Control. *Therapeutic Innovation & Regulatory Science*, *57*(3), 611–618. https://doi.org/10.1007/s43441-023-00497-2

Loiseau, N., Trichelair, P., He, M., Andreux, M., Zaslavskiy, M., Wainrib, G., & Blum, M. G. B. (2022). External control arm analysis: An evaluation of propensity score approaches, G-computation, and doubly debiased machine learning. *BMC Medical Research Methodology*, *22*(1), 335. https://doi.org/10.1186/s12874-022-01799-z

Martin, T., Krishnan, A., Yong, K., Weisel, K., Mehra, M., Nair, S., Qi, K., Londhe, A., Diels, J., Crivera, C., Jackson, C. C., Olyslager, Y., Vogel, M., Schecter, J. M., Banerjee, A., Valluri, S., Usmani, S. Z., Berdeja, J. G., & Jagannath, S. (2022). Comparative effectiveness of ciltacabtagene autoleucel in CARTITUDE‐1 versus physician’s choice of therapy in the Flatiron Health multiple myeloma cohort registry for the treatment of patients with relapsed or refractory multiple myeloma. *eJHaem*, *3*(1), 97–108. https://doi.org/10.1002/jha2.312

Mateos, M.-V., Chari, A., Usmani, S. Z., Goldschmidt, H., Weisel, K., Qi, K., Londhe, A., Nair, S., Lin, X., Pei, L., Ammann, E., Kobos, R., Smit, J., Parekh, T., Marshall, A., Slavcev, M., & Moreau, P. (2023). Comparative Efficacy of Teclistamab Versus Physician’s Choice of Therapy in the Long-term Follow-up of APOLLO, POLLUX, CASTOR, and EQUULEUS Clinical Trials in Patients With Triple-class Exposed Relapsed or Refractory Multiple Myeloma. *Clinical Lymphoma Myeloma and Leukemia*, *23*(5), 385–393. https://doi.org/10.1016/j.clml.2023.02.006

McMahon, P. M., Kong, C. Y., Johnson, B. E., Weinstein, M. C., Weeks, J. C., Kuntz, K. M., Shepard, J.-A. O., Swensen, S. J., & Gazelle, G. S. (2008). Estimating long-term effectiveness of lung cancer screening in the Mayo CT screening study. *Radiology*, *248*(1), 278–287. https://doi.org/10.1148/radiol.2481071446

Mehtälä, J., Ali, M., Miettinen, T., Partanen, L., Laapas, K., Niemelä, P. T., Khorlo, I., Ström, S., Kurki, S., Vapalahti, J., Abdelgawwad, K., & Leinonen, J. V. (2023). Utilization of anonymization techniques to create an external control arm for clinical trial data. *BMC Medical Research Methodology*, *23*(1), 258. https://doi.org/10.1186/s12874-023-02082-5

Menefee, M. E., Gong, Y., Mishra-Kalyani, P. S., Sridhara, R., Kanapuru, B., Blumenthal, G. M., & Pazdur, R. (2019). Project Switch: Docetaxel as a potential synthetic control in metastatic non-small cell lung cancer (mNSCLC) trials. *Journal of Clinical Oncology*, *37*(15_suppl), 9105–9105. https://doi.org/10.1200/JCO.2019.37.15_suppl.9105

Mishra-Kalyani, P. S., Amiri Kordestani, L., Rivera, D. R., Singh, H., Ibrahim, A., DeClaro, R. A., Shen, Y., Tang, S., Sridhara, R., Kluetz, P. G., Concato, J., Pazdur, R., & Beaver, J. A. (2022). External control arms in oncology: Current use and future directions. *Annals of Oncology*, *33*(4), 376–383. https://doi.org/10.1016/j.annonc.2021.12.015

Moreau, P., Mateos, M.-V., Gonzalez Garcia, M. E., Einsele, H., De Stefano, V., Karlin, L., Lindsey-Hill, J., Besemer, B., Vincent, L., Kirkpatrick, S., Delforge, M., Perrot, A., van de Donk, N. W. C. J., Pawlyn, C., Manier, S., Leleu, X., Martinez-Lopez, J., Ghilotti, F., Diels, J., … Weisel, K. (2024). Comparative Effectiveness of Teclistamab Versus Real-World Physician’s Choice of Therapy in LocoMMotion and MoMMent in Triple-Class Exposed Relapsed/Refractory Multiple Myeloma. *Advances in Therapy*, *41*(2), 696–715. https://doi.org/10.1007/s12325-023-02738-0

Moreau, P., van de Donk, N. W. C. J., Delforge, M., Einsele, H., De Stefano, V., Perrot, A., Besemer, B., Pawlyn, C., Karlin, L., Manier, S., Leleu, X., Weisel, K., Ghilotti, F., Diels, J., Elsada, A., Morano, R., Strulev, V., Pei, L., Kobos, R., … Mateos, M.-V. (2023). Comparative Efficacy of Teclistamab Versus Current Treatments in Real-World Clinical Practice in the Prospective LocoMMotion Study in Patients with Triple-Class-Exposed Relapsed and/or Refractory Multiple Myeloma. *Advances in Therapy*, *40*(5), 2412–2425. https://doi.org/10.1007/s12325-023-02480-7

Murray, T. A., Thall, P. F., Schortgen, F., Asfar, P., Zohar, S., & Katsahian, S. (2021). Robust Adaptive Incorporation of Historical Control Data in a Randomized Trial of External Cooling to Treat Septic Shock. *Bayesian Analysis*, *16*(3), 825–844. https://doi.org/10.1214/20-ba1229

Myles, P., Ordish, J., & Tucker, A. (2023). The potential synergies between synthetic data and in silico trials in relation to generating representative virtual population cohorts. *Progress in Biomedical Engineering*, *5*(1), 013001. https://doi.org/10.1088/2516-1091/acafbf

Narita, Y., Yoshimoto, T., Namai, T., Asakawa, T., Kawakami, S., Gower-Page, C., Reyes-Rivera, I., Patel, A., & Nakamura, Y. (2022). Pertuzumab Plus Trastuzumab for Treatment-Refractory HER2-Amplified Metastatic Colorectal Cancer: Comparison of the MyPathway Trial With a Real-World External Control Arm. *JCO Clinical Cancer Informatics*, *6*, e2200022. https://doi.org/10.1200/CCI.22.00022

Neehal, N., Anand, V., & Bennett, K. P. (2023). Framework for Research in Equitable Synthetic Control Arms. *AMIA ... Annual Symposium Proceedings. AMIA Symposium*, *2023*, 530–539.

Nicholson, K., Chan, J., Macklin, E. A., Levine-Weinberg, M., Breen, C., Bakshi, R., Grasso, D. L., Wills, A.-M., Jahandideh, S., Taylor, A. A., Beaulieu, D., Ennist, D. L., Andronesi, O., Ratai, E.-M., Schwarzschild, M. A., Cudkowicz, M., & Paganoni, S. (2018). Pilot trial of inosine to elevate urate levels in amyotrophic lateral sclerosis. *Annals of Clinical and Translational Neurology*, *5*(12), 1522–1533. https://doi.org/10.1002/acn3.671

Noguer, J., Contreras, I., Mujahid, O., Beneyto, A., & Vehi, J. (2022). Generation of Individualized Synthetic Data for Augmentation of the Type 1 Diabetes Data Sets Using Deep Learning Models. *Sensors*, *22*(13), 4944. https://doi.org/10.3390/s22134944

O’Haire, S., Degeling, K., Franchini, F., Tran, B., Luen, S. J., Gaff, C., Smith, K., Fox, S., Desai, J., & IJzerman, M. (2022). Comparing Survival Outcomes for Advanced Cancer Patients Who Received Complex Genomic Profiling Using a Synthetic Control Arm. *Targeted Oncology*, *17*(5), 539–548. https://doi.org/10.1007/s11523-022-00910-0

Orbach, D., Carton, M., Khadir, S. K., Feuilly, M., Kurtinecz, M., Phil, D., Vokuhl, C., Koscielniak, E., Pierron, G., Lemelle, L., & Sparber-Sauer, M. (2024). Therapeutic benefit of larotrectinib over the historical standard of care in patients with locally advanced or metastatic infantile fibrosarcoma (EPI VITRAKVI study). *ESMO Open*, *9*(5), 103006. https://doi.org/10.1016/j.esmoop.2024.103006

Polito, L., Liang, Q., Pal, N., Mpofu, P., Sawas, A., Humblet, O., Rufibach, K., & Heinzmann, D. (2024). Applying the estimand and target trial frameworks to external control analyses using observational data: A case study in the solid tumor setting. *Frontiers in Pharmacology*, *15*, 1223858. https://doi.org/10.3389/fphar.2024.1223858

Polley, M.-Y. C., Schwartz, D., Karrison, T., & Dignam, J. J. (2024). Leveraging external control data in the design and analysis of neuro-oncology trials: Pearls and perils. *Neuro-Oncology*, *26*(5), 796–810. https://doi.org/10.1093/neuonc/noae005

Popat, S., Liu, S. V., Scheuer, N., Hsu, G. G., Lockhart, A., Ramagopalan, S. V., Griesinger, F., & Subbiah, V. (2022). Addressing challenges with real-world synthetic control arms to demonstrate the comparative effectiveness of Pralsetinib in non-small cell lung cancer. *Nature Communications*, *13*(1), 3500. https://doi.org/10.1038/s41467-022-30908-1

Rudrapatna, V. A., Cheng, Y.-W., Feuille, C., Mosenia, A., Shih, J., Shi, Y., Roberson, O., Rubin, B., Butte, A. J., Mahadevan, U., Skomrock, N., Erondu, N., Chehoud, C., Rahim, S., Apfel, D., Curran, M., Khan, N. S., O’Brien, C., Terry, N., & Martini, B. D. (2023). Creation of an ustekinumab external control arm for Crohn’s disease using electronic health records data: A pilot study. *PloS One*, *18*(3), e0282267. https://doi.org/10.1371/journal.pone.0282267

Schneeweiss, S. (2024). Enhancing External Control Arm Analyses through Data Calibration and Hybrid Designs. *Clinical Pharmacology and Therapeutics*. https://doi.org/10.1002/cpt.3364

Schröder, C., Lawrance, M., Li, C., Lenain, C., Mhatre, S. K., Fakih, M., Reyes-Rivera, I., & Bretscher, M. T. (2021). Building External Control Arms From Patient-Level Electronic Health Record Data to Replicate the Randomized IMblaze370 Control Arm in Metastatic Colorectal Cancer. *JCO Clinical Cancer Informatics*, *5*, 450–458. https://doi.org/10.1200/CCI.20.00149

Seeger, J. D., Davis, K. J., Iannacone, M. R., Zhou, W., Dreyer, N., Winterstein, A. G., Santanello, N., Gertz, B., & Berlin, J. A. (2020). Methods for external control groups for single arm trials or long‐term uncontrolled extensions to randomized clinical trials. *Pharmacoepidemiology and Drug Safety*, *29*(11), 1382–1392. https://doi.org/10.1002/pds.5141

Sengupta, S., Ntambwe, I., Tan, K., Liang, Q., Paulucci, D., Castellanos, E., Fiore, J., Lane, S., Micsinai Balan, M., Viraswami‐Apanna, K., Sethuraman, V., Samant, M., & Tiwari, R. (2023). Emulating Randomized Controlled Trials with Hybrid Control Arms in Oncology: A Case Study. *Clinical Pharmacology & Therapeutics*, *113*(4), 867–877. https://doi.org/10.1002/cpt.2841

Serrano, C., Rothschild, S., Villacampa, G., Heinrich, M. C., George, S., Blay, J.-Y., Sicklick, J. K., Schwartz, G. K., Rastogi, S., Jones, R. L., Rutkowski, P., Somaiah, N., Navarro, V., Evans, D., & Trent, J. C. (2023). Rethinking placebos: Embracing synthetic control arms in clinical trials for rare tumors. *Nature Medicine*, *29*(11), 2689–2692. https://doi.org/10.1038/s41591-023-02578-z

Silva, P., Janjan, N., Ramos, K. S., Udeani, G., Zhong, L., Ory, M. G., & Smith, M. L. (2023). External control arms: COVID-19 reveals the merits of using real world evidence in real-time for clinical and public health investigations. *Frontiers in Medicine*, *10*, 1198088. https://doi.org/10.3389/fmed.2023.1198088

Siu, D. H. W., Lin, F. P. Y., Cho, D., Lord, S. J., Heller, G. Z., Simes, R. J., & Lee, C. K. (2024). Framework for the Use of External Controls to Evaluate Treatment Outcomes in Precision Oncology Trials. *JCO Precision Oncology*, *8*, e2300317. https://doi.org/10.1200/PO.23.00317

Stanford University. (2023). *Virtual Control Arms for Clinical Trials using Deep Learning*. https://techfinder.stanford.edu/technology/virtual-control-arms-clinical-trials-using-deep-learning

Strayhorn, J. M. (2021). Virtual controls as an alternative to randomized controlled trials for assessing efficacy of interventions. *BMC Medical Research Methodology*, *21*(1), 3. https://doi.org/10.1186/s12874-020-01191-9

Struebing, A., McKibbon, C., Ruan, H., Mackay, E., Dennis, N., Velummailum, R., He, P., Tanaka, Y., Xiong, Y., Springford, A., & Rosenlund, M. (2024). Augmenting external control arms using Bayesian borrowing: A case study in first-line non-small cell lung cancer. *Journal of Comparative Effectiveness Research*, *13*(5), e230175. https://doi.org/10.57264/cer-2023-0175

Suissa, S. (2021). Single-arm Trials with Historical Controls: Study Designs to Avoid Time-related Biases. *Epidemiology*, *32*(1), 94–100. https://doi.org/10.1097/EDE.0000000000001267

Switchenko, J. M., Heeke, A. L., Pan, T. C., & Read, W. L. (2019). The use of a predictive statistical model to make a virtual control arm for a clinical trial. *PLOS ONE*, *14*(9), e0221336. https://doi.org/10.1371/journal.pone.0221336

Tan, W. K., Segal, B. D., Curtis, M. D., Baxi, S. S., Capra, W. B., Garrett-Mayer, E., Hobbs, B. P., Hong, D. S., Hubbard, R. A., Zhu, J., Sarkar, S., & Samant, M. (2022). Augmenting control arms with real-world data for cancer trials: Hybrid control arm methods and considerations. *Contemporary Clinical Trials Communications*, *30*, 101000. https://doi.org/10.1016/j.conctc.2022.101000

Thorlund, K., Dron, L., Park, J. J., & Mills, E. J. (2020). Synthetic and External Controls in Clinical Trials – A Primer for Researchers. *Clinical Epidemiology*, *Volume 12*, 457–467. https://doi.org/10.2147/CLEP.S242097

Thorlund, K., Duffield, S., Popat, S., Ramagopalan, S., Gupta, A., Hsu, G., Arora, P., & Subbiah, V. (2024). Quantitative bias analysis for external control arms using real-world data in clinical trials: A primer for clinical researchers. *Journal of Comparative Effectiveness Research*, *13*(3), e230147. https://doi.org/10.57264/cer-2023-0147

Uemura, Y., Ozaki, R., Shinozaki, T., Ohtsu, H., Shimizu, Y., Izumi, K., Saito, S., Matsunaga, N., & Ohmagari, N. (2023). Comparative effectiveness of tocilizumab vs standard care in patients with severe COVID-19-related pneumonia: A retrospective cohort study utilizing registry data as a synthetic control. *BMC Infectious Diseases*, *23*(1), 849. https://doi.org/10.1186/s12879-023-08840-6

Umer, F., & Adnan, N. (2024). Generative artificial intelligence: Synthetic datasets in dentistry. *BDJ Open*, *10*(1), 13. https://doi.org/10.1038/s41405-024-00198-4

Van Le, H., Van Naarden Braun, K., Nowakowski, G. S., Sermer, D., Radford, J., Townsend, W., Ghesquieres, H., Menne, T., Porpaczy, E., Fox, C. P., Schusterbauer, C., Liu, F. F., Yue, L., De Benedetti, M., & Hasskarl, J. (2023). Use of a real-world synthetic control arm for direct comparison of lisocabtagene maraleucel and conventional therapy in relapsed/refractory large B-cell lymphoma. *Leukemia & Lymphoma*, *64*(3), 573–585. https://doi.org/10.1080/10428194.2022.2160200

Velummailum, R. R., McKibbon, C., Brenner, D. R., Stringer, E. A., Ekstrom, L., & Dron, L. (2023). Data Challenges for Externally Controlled Trials: Viewpoint. *Journal of Medical Internet Research*, *25*, e43484. https://doi.org/10.2196/43484

Visentin, R., Dalla Man, C., Kovatchev, B., & Cobelli, C. (2014). The University of Virginia/Padova Type 1 Diabetes Simulator Matches the Glucose Traces of a Clinical Trial. *Diabetes Technology & Therapeutics*, *16*(7), 428–434. https://doi.org/10.1089/dia.2013.0377

Walker, B., Ray, H. E., Shao, P., D’Ambrosio, C., White, C., & Walker, M. S. (2024). Comparing prospectively assigned trial and real-world lung cancer patients. *Journal of Comparative Effectiveness Research*, *13*(7), e230176. https://doi.org/10.57264/cer-2023-0176

Wang, X., Dormont, F., Lorenzato, C., Latouche, A., Hernandez, R., & Rouzier, R. (2023). Current perspectives for external control arms in oncology clinical trials: Analysis of EMA approvals 2016–2021. *Journal of Cancer Policy*, *35*, 100403. https://doi.org/10.1016/j.jcpo.2023.100403

Wei, W., Zhang, Y., Roychoudhury, S., & Alzheimer’s Disease Neuroimaging Initiative. (2024). Propensity score weighted multi-source exchangeability models for incorporating external control data in randomized clinical trials. *Statistics in Medicine*. https://doi.org/10.1002/sim.10158

Yin, X., Davi, R., Lamont, E. B., Thaker, P. H., Bradley, W. H., Leath, C. A., Moore, K. M., Anwer, K., Musso, L., & Borys, N. (2023). Historic Clinical Trial External Control Arm Provides Actionable GEN-1 Efficacy Estimate Before a Randomized Trial. *JCO Clinical Cancer Informatics*, *7*, e2200103. https://doi.org/10.1200/CCI.22.00103

Yin, X., Mishra-Kalyan, P. S., Sridhara, R., Stewart, M. D., Stuart, E. A., & Davi, R. C. (2022). Exploring the Potential of External Control Arms created from Patient Level Data: A case study in non-small cell lung cancer. *Journal of Biopharmaceutical Statistics*, *32*(1), 204–218. https://doi.org/10.1080/10543406.2021.2011901

Yoshino, T., Shi, Q., Misumi, T., Bando, H., Wakabayashi, M., Raeisi, M., Andre, T., & De Gramont, A. (2023). A synthetic control arm for refractory metastatic colorectal cancer: The no placebo initiative. *Nature Medicine*, *29*(10), 2389–2390. https://doi.org/10.1038/s41591-023-02488-0

Zayadi, A., Edge, R., Parker, C. E., Macdonald, J. K., Neustifter, B., Chang, J., Zhong, G., Singh, S., Feagan, B. G., Ma, C., & Jairath, V. (2023). Use of external control arms in immune-mediated inflammatory diseases: A systematic review. *BMJ Open*, *13*(12), e076677. https://doi.org/10.1136/bmjopen-2023-076677

Zhou, T., & Ji, Y. (2021). Incorporating external data into the analysis of clinical trials via Bayesian additive regression trees. *Statistics in Medicine*, *40*(28), 6421–6442. https://doi.org/10.1002/sim.9191

Zhu, J., & Tang, R. S. (2022). A proper statistical inference framework to compare clinical trial and real-world progression-free survival data. *Statistics in Medicine*, *41*(29), 5738–5752. https://doi.org/10.1002/sim.9590

Zou, K. H., Vigna, C., Talwai, A., Jain, R., Galaznik, A., Berger, M. L., & Li, J. Z. (2024). The Next Horizon of Drug Development: External Control Arms and Innovative Tools to Enrich Clinical Trial Data. *Therapeutic Innovation & Regulatory Science*, *58*(3), 443–455. https://doi.org/10.1007/s43441-024-00627-4
